# Habitat discrimination by gravid *Anopheles gambiae sensu lato* – a push-pull system

**DOI:** 10.1186/1475-2875-13-133

**Published:** 2014-04-02

**Authors:** Manuela Herrera-Varela, Jenny Lindh, Steven W Lindsay, Ulrike Fillinger

**Affiliations:** 1 Department of Diseases Control, London School of Hygiene and Tropical Medicine, London, UK; 2 International Centre for Insect Physiology and Ecology (icipe)-Thomas Odhiambo Campus, Mbita, Kenya; 3 Royal Institute of Technology, Stockholm, Sweden; 4 School of Biological and Biomedical Sciences, Durham University, Durham, UK

**Keywords:** Oviposition site selection, *Anopheles gambiae*, Natural infusion, Olfactory memory

## Abstract

**Background:**

The non-random distribution of anopheline larvae in natural habitats suggests that gravid females discriminate between habitats of different quality. Whilst physical and chemical cues used by *Culex* and *Aedes* vector mosquitoes for selecting an oviposition site have been extensively studied, those for *Anopheles* remain poorly explored. Here the habitat selection by *Anopheles gambiae sensu lato (s.l.)*, the principal African malaria vector, was investigated when presented with a choice of two infusions made from rabbit food pellets, or soil.

**Methods:**

Natural colonization and larval survival was evaluated in artificial ponds filled randomly with either infusion. Dual-choice, egg-count bioassays evaluated the responses of caged gravid females to (1) two- to six-day old infusions *versus* lake water; (2) autoclaved *versus* non-autoclaved soil infusions; and assessed (3) the olfactory memory of gravid females conditioned in pellet infusion as larvae.

**Results:**

Wild *Anopheles* exclusively colonized ponds with soil infusion and avoided those with pellet infusion. When the individual infusions were tested in comparison with lake water, caged *An. gambiae sensu stricto (s.s.)* showed a dose response: females increasingly avoided the pellet infusion with increasing infusion age (six-day *versus* lake water: odds ratio (OR) 0.22; 95% confidence interval (CI) 0.1-0.5) and showed increasing preference to lay eggs as soil infusion age increased (six-day *versus* lake water: OR 2.1; 95% CI 1.4-3.3). Larvae survived in soil infusions equally well as in lake water but died in pellet infusions. *Anopheles gambiae s.s.* preferred to lay eggs in the non-autoclaved soil (OR 2.6; 95% CI 1.8-3.7) compared with autoclaved soil. There was no change in the avoidance of pellet infusion by individuals reared in the infusion compared with those reared in lake water.

**Conclusion:**

Wild and caged *An. gambiae s.l.* females discriminate between potential aquatic habitats for oviposition. These choices benefit the survival of the offspring. Although the study was not designed to distinguish between stimuli that acted over a distance or on contact, it could be demonstrated that the choice of habitat is mediated by chemical cues based on both preference and avoidance. These cues, if identified, might be developed for ‘push-pull’ strategies to improve malaria vector monitoring and control.

## Background

Selection of suitable oviposition sites is a critical step in the life history of mosquitoes [1]. This is a process whereby individuals select and occupy a non-random set of aquatic habitats. Habitat selection is of major importance for the interpretation of spatial and temporal distributions of populations, and for understanding intra and inter-specific relations that influence the abundance of individuals [2,3]. Organisms without any parental care are likely to choose habitats based on a set of innate or learned cues in order to maximize the survival and fitness of their offspring [2,4].

Mosquitoes utilize a wide range of aquatic niches for oviposition, including natural ponds, puddles, stream fringes, marshes, tree-holes and plant axils, man-made pits, drains, rice fields, and containers [5]. Field studies have shown that mosquitoes are discriminating in selecting sites for egg deposition [6,7] and that oviposition choices made by gravid females are a key factor in determining larval distribution [8-11]. Although different species are found in the same type of habitat, oviposition site selectivity is considerably species specific [12]. Immature stages of *Anopheles gambiae sensu lato (s.l.)*, the major malaria vector in sub-Saharan Africa, are typically described as inhabiting very small, temporary sunlit pools and puddles without vegetation [13-16]. However, reviews of the literature and recent research on larval ecology have shown that this is a gross oversimplification of the wide range of habitats colonized by this species [17-19], a fact recognized over half a century ago by Holstein who reviewed the ‘extraordinary diversity of the breeding places’ of *An. gambiae s.l.*[19] . Numerous studies have described how the presence of *An. gambiae s.l.* larvae [17,20-23] and the capacity of individual habitats for generating adult mosquitoes [6,8,17,24,25] differs markedly over space and time, yet these surveys failed to reveal any risk factors that could consistently predict sites preferred by *An. gambiae s.l.*[17,20-23]. This might lead to the conclusion that this species randomly deposits its eggs in a large range of habitats and that the heterogeneous distribution of larvae results from the survival of larvae in the aquatic habitat [9,10] rather than the adults’ choice.

Surprisingly, fully gravid malaria vectors looking for suitable larval habitats have been grossly understudied [26]. Compared to the wealth of knowledge of the physical and chemical factors used by gravid culicine for selecting an oviposition site [27-40] those potentially used by the world’s most deadly malaria vector remain almost unexplored. Whereas many publications recognize that the distribution of larvae between seemingly suitable aquatic habitats is probably due to the choice of the gravid female [6,8,24,41-43] and that this choice probably impacts on the fitness of her offspring, there is little empirical evidence to support these assertions. Most recent research has evaluated the characteristics of aquatic habitats associated with the presence and absence of larvae [15,21,44-46] but the understanding of the behaviour of gravid female *An. gambiae s.l.* when searching for an oviposition site remains, at best, sketchy [8,41,47-53].

Laboratory studies demonstrated that physical conditions of the aquatic habitats influence oviposition site selection in *An. gambiae s.l.,* with females preferring dark backgrounds to pale ones, muddy water to clear water and fully hydrated substrates [8,41,50,54]. Turbidity has been suggested as an important physical cue for oviposition behaviour in *An. gambiae s.l.* although the evidence for this is contradictory [55,56].

Even less is known about the chemical cues and their interaction with physical factors. Water vapour is itself an attractant to gravid mosquitoes [57]. It has been shown that gravid *An. gambiae s.l.* are sensitive to bacteria-derived odours [47,53,58] which have been associated with increased [47,53] and reduced [58] egg numbers compared to sterile media in cage bioassays. Whilst over 20 putative oviposition semiochemicals have been suggested in the literature, based on the analyses of bacteria- or habitat-derived volatile chemicals and electro-antennogram studies [53,59], there is only one report [60] of two chemicals inducing a behavioural response in gravid females (one increasing and one decreasing the oviposition response).

Here the oviposition behaviour of *An. gambiae s.l.* was explored to test the hypotheses that a gravid *An. gambiae s.l.* female evaluates the suitability of a habitat using chemical cues from water bodies that oviposition choices made by a gravid female benefit the offspring and that this choice cannot be modified by experience in one generation.

Habitat selection by gravid *An. gambiae s.l.* was tested by presenting a choice of two infusions; one made with soil from an area where natural habitats occur frequently, and one made with rabbit food pellets. Rabbit food pellets are frequently used as diet for mosquito larvae in insectaries [61,62] and infusions made of grass, hay and other plant material, including rabbit food pellets have shown to be attractive to a range of mosquito species and have been used in gravid traps [63-66]. The aim was to explore whether *Anopheles gambiae s.l.* might also be drawn to this infusion.

Natural colonization and larval survival was evaluated in artificial ponds filled randomly with either infusion. As a consequence of the field observations, two-choice, egg-count bioassays were used to explore pattern of oviposition seen in the field. Experiments were designed to address the following questions: 1) Do gravid *An. gambiae s.l.* females discriminate between different habitats when searching for an oviposition site? 2) Does the oviposition choice benefit the survival of their offspring? 3) Are gravid females guided by preference (attractants/stimulants) or avoidance (repellents/deterrents)? 4) Are oviposition choices likely to be based on chemical cues? and, 5) Is the choice made by a gravid female influenced by her olfactory memory of her larval habitat?

## Methods

### Study site

Experiments were carried out at the International Centre for Insect Physiology and Ecology (icipe), Mbita, on the shores of Lake Victoria, Western Kenya (geographic coordinates 0°26′06.19″ South; 34°12′53.13″ East; altitude 1,137 m above sea level). Mbita has a typical tropical climate; temperatures oscillate between 18-28°C and there is annual rainfall of 1,436 mm (based on data from icipe meteorological station for 2010–2012). Two rainy seasons occur annually, the long rainy season between March and June and the short rainy season between October and December. Malaria is endemic in the area and transmitted by three vectors, which are in order of their abundance: *Anopheles arabiensis*, *Anopheles gambiae sensu stricto (s.s.)* and *Anopheles funestus*[67].

### Mosquitoes

Open-field trials were conducted with wild anopheline and culicine females that oviposited in tubs of water sunk into the ground. These were colonized within three days. Laboratory experiments were carried out with insectary-reared *An. gambiae s.s*. (Mbita strain) supplied by icipe’s insectary and reared following standard operating procedures. Briefly, larvae were reared in round plastic tubs (diameter 0.6 m) filled with water from Lake Victoria and fed Tetramin® fish food twice daily. Larvae were collected randomly from several tubs on the day of experiment. Gravid mosquitoes were prepared by selecting 300 female and 300 male mosquitoes, two to three days old, from their rearing cages at 12.00 and keeping them in 30 × 30 × 30 cm netting cages at 25-28°C and 68-75% relative humidity. To avoid mosquito desiccation, cotton towels (folded to 25×12 cm) were saturated with lake water and placed over the cages. Mosquitoes were starved of sugar for seven hours before blood feeding and allowed to feed on a human arm for 15 min at 19.00 on the same day. After feeding, mosquitoes were provided with 6% glucose solution *ad libitum.* This procedure was repeated 24 hours later. After the first blood meal unfed female mosquitoes were removed from the cages. Fed female mosquitoes were kept together with males for two days after the second blood meal before using them in an experiment (i e, females four to five days after first blood meal). In the afternoon (16.30) of the day of an experiment 45–100 (depending on experiment and availability) visually presumed gravid females, that is with an enlarged, pale white abdomen, were selected from the holding cage. A small percentage of these mosquitoes were probably not gravid because most females needed two blood meals to reach full gravidity and some never reach full gravidity even after three feeds [68,69]. Whilst two meals were provided it cannot be guaranteed that two meals were taken by all females. This might be the reason that not all mosquitoes exposed to oviposition medium in experiments laid eggs (responded), therefore the number of responders was smaller than the number tested. Non-responders were excluded from the analyses.

## Experimental procedures

### Do gravid *Anopheles gambiae sensu lato* females discriminate between different habitats when searching for an oviposition site?

To explore natural colonization of habitats by wild mosquitoes, 20 artificial habitats were created by implanting 20 plastic tubs (40 cm diameter, 20 cm deep) into an open-sunlit field during the long rainy season in May 2011. The tubs were placed in four lines of five tubs each 4 m apart [70]. Two different substrates were randomly offered in the artificial habitats. Half of the tubs (ten) received 30 g of rabbit food pellets (Scooby® rabbit and rodent food, Nairobi) containing hay and grains from maize, wheat, barley, cotton, sunflower, soya bean meal, and traces of molasses, vitamins and minerals. The remaining half of the tubs (ten) received 2 kg of dry soil taken from a field at icipe. Soil texture was characterized as a silty clay loam according to the US Department of Agriculture (USDA) texture triangle [71] using the detergent method [72] to separate and quantify soil mineral particles of different size. A volume of 15 l of lake water pumped from Lake Victoria, was added to each tub and the water level was held constant by adding water to the 15 l mark daily. The two treatments are henceforth referred to as pellet and soil infusion. To study the oviposition response of wild mosquitoes the tubs were monitored daily between 08.00 and 10.00 by dipping five times per tub with a standard dipper (350 ml). Two different dippers were used for the two treatments to avoid contamination. Four dips were taken from the edge of the tubs and one from the middle. The content of each dip was emptied into a white plastic bowl and all early instars (first and second stage larvae) counted and recorded for both culicine and anopheline mosquitoes. All larvae and the water were returned to the respective tub. The tubs were followed for 16 days. Ponds were searched for pupae and collected daily to prevent any emergence of potential disease vectors. Pupae were allowed to emerge in cages in the laboratory and any anophelines emerging identified to species using morphological keys [13,73] and for specimens of the *An. gambiae* complex using the ribosomal DNA-polymerase chain reaction (PCR) method to distinguish between the two local species of the complex *An. gambiae* s.s. and *An. arabiensis*[74].

### Does the oviposition choice benefit the survival of their offspring?

Larval survival was assessed by introducing individual, insectary-reared, first instar *An. gambiae s.s.* larvae in infusions collected from the tubs set up in the field. Infusion samples were taken after one, six, 11, and 16 days. One-hundred ml of infusion was collected from each of the ten tubs per treatment and pooled per treatment (soil or pellet infusion) in a plastic bottle. Lake water was used as a control. First instars were introduced in 100 ml plastic cups containing 50 ml of pellet infusion, soil infusion or lake water. Twenty larvae were exposed individually per treatment and collection day. Larvae were fed every second day with finely ground Tetramin® Baby fish food. Food was provided with a blunt toothpick that was first wetted in lake water and then dipped quickly, not more than 1 mm deep into the ground food, and then dipped onto the surface of the test water. Larval development was monitored daily and the time of death or time to pupation and emergence recorded. This experiment was implemented under ambient conditions in a semi-field system (80 sq m) with screened walls and a glass roof [75].

### Are gravid females guided by preferences or avoidance?

Based on the analysis of the field data a series of two-choice, egg-count bioassays were designed to investigate if the response of wild gravid females observed in the field was based on avoidance or preference of an infusion or both.

**Figure 1:**
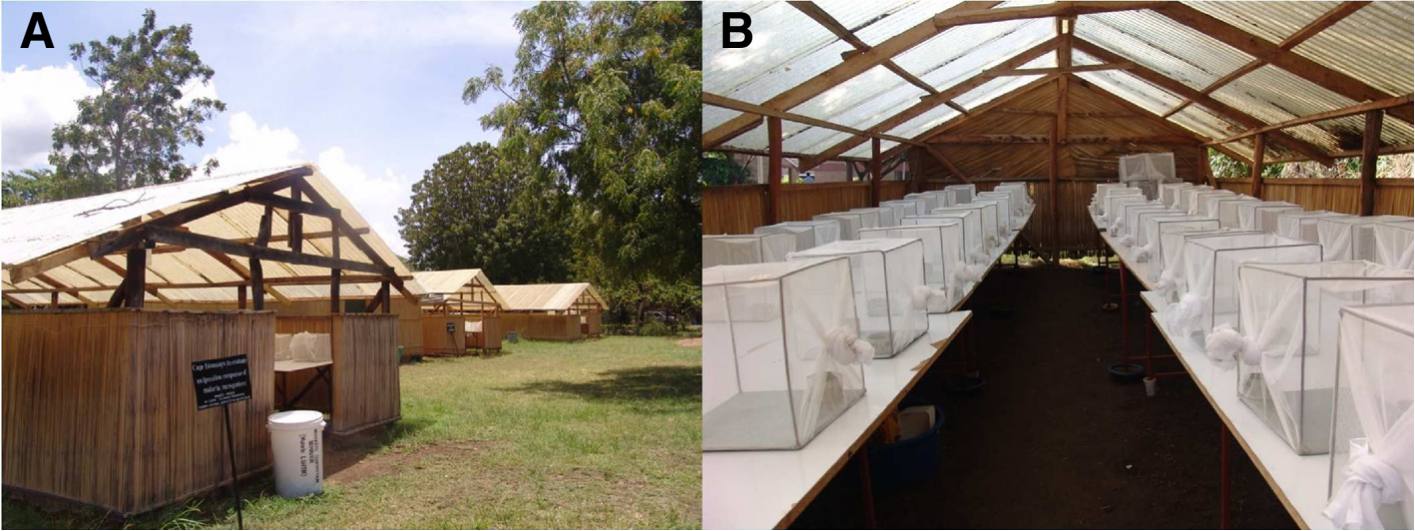
**Location of egg-count bioassays. (A)** Sheds (10 m long × 5 m wide × 2.8 m high) with walls made of reed mats and a roof made of translucent corrugated polycarbonate sheets. **(B)** Interior of a shed. In each shed two tables hold up to 25 standard cages each, allowing 40 cm of space between adjacent cages.

Gravid females were selected from insectary cages and transferred individually to 30×30×30 cm cages. In each cage two glass cups (Pyrex®, 100 ml, 70 mm diameter), surrounded by tightly fitting aluminium cylinders, so that mosquitoes could see only the water surface, were filled with 100 ml of either the control or test medium and placed in diagonal corners of the cage. Prior to use, cups and cylinders were cleaned with detergent, then autoclaved and kept in an oven at 200°C for at least two hours. The position of oviposition cups containing the test medium was alternated between adjacent cages to control for possible position effect. The placement of the first test cup was randomly allocated for one of the four cage corners in the first cage. Subsequent test cups were rotated in the next possible corners in a clockwise direction relative to the position of the preceding cup. One control cup was added in each cage diagonal to the test cup to complete a two-choice set up. The experiments were carried out in makeshift sheds (Figure 1) that exposed the mosquitoes to ambient light, temperature and relative humidity but protected the cages from rain.

Two sets of experiments were carried out consecutively (Table 1, Set 1 and 2). In the first set oviposition choice was evaluated for two-, four- and six-day old pellet infusions compared with lake water. In the second set, the oviposition choice was evaluated for two, four and six-day old soil infusions compared with lake water. In both sets of experiments internal controls were used to validate the two-choice experiment. Here equal numbers of cages were set up where both cups in a cage contained lake water and were labelled randomly as control and test cup, assuming that gravid females lay eggs in both cups in an equal proportion.

**Table 1:** **Summary details of dual-choice, egg-count bioassays to evaluate oviposition choices in *Anopheles gambiae sensu stricto***

**Dual-choice cage, egg-count bioassays**	**Treatments**		**No of rounds**	**Total no of females responding for all rounds (total number set up)**
	**Control**	**Test**		
Set 1: Pellet infusions	Lake water	Lake water	3	66 (75)
	Lake water	2-day old pellet infusion		64 (75)
	Lake water	4-day old pellet infusion		67 (75)
	Lake water	6-day old pellet infusion		68 (75)
Set 2: Soil infusions	Lake water	Lake water	9	153 (225)
	Lake water	2-day old soil infusion		161 (225)
	Lake water	4-day old soil infusion		160 (225)
	Lake water	6-day old soil infusion		171 (225)
Set 3: Vision *versus* olfaction in soil infusions	Lake water	Lake water	12	186 (220)
	Lake water	6-day old soil infusion		150 (220)
	Lake water	Autoclaved 6-day old soil infusion		157 (220)
	Autoclaved 6-day old soil infusion	6-day old soil infusion		169 (220)
Set 4: Olfactory memory – pellet infusions	**Lake water reared *An. gambiae *** **females**			
	Lake water	6 day old pellet infusion	1	31 (45)
	**Pellet infusion reared *An. gambiae *** **females**			
	Lake water	6-day old pellet infusion	1	37 (45)

Infusions were prepared in a similar way as for the field tests. Fifteen l of lake water were either incubated with 30 g of pellets to prepare a pellet infusion or incubated with 2 kg of soil to prepare a soil infusion. Infusions were prepared in a plastic tub (40 cm diameter 20 cm depth) six days, four days and two days before the day of experiment in order to test all ages in parallel. Tubs were covered with mosquito netting and kept in makeshift sheds at ambient conditions but protected from rain. Experiments were implemented over three to nine rounds depending on the availability of gravid females and the response rate per round (Table 1) with fresh batches of infusions and different batches of mosquitoes for every round. On the day of experiment infusions were sieved through a clean piece of cotton cloth to remove large debris remaining from the pellets or soil.

Fifteen to 25 replicate cages per treatment were set up per round. A single gravid female was introduced per cage at 17.30. The next morning between 08.00 and 09.00 the absence or presence and the number of eggs was recorded for the control and test cup in each cage.

Turbidity, conductivity, dissolved oxygen, and pH were measured in five cups per treatment in four different batches of pellet, soil infusions and lake water using a turbidity meter (TURB 355IR, WTW Germany) and a multimeter (Multi 340i, WTW, Germany). In addition one batch of pellet, soil infusion and lake water was tested for ammonium (NH_4_+), carbonate hardness, total hardness, nitrate (NO_3_^−^), nitrite (NO_2_^−^), and phosphate (PO_4_^3−^) content using Aquamerck® test kits from the compact laboratory for water testing (Aquamerck® No.111151, Germany).

### Are oviposition choices likely to be based on chemical cues?

Soil infusions differed strongly in colour and turbidity from lake water. To assess if the oviposition response observed was based on visual or chemical cues, a third set of dual-choice, egg-count bioassays were implemented with six-day old soil infusions (Table 1, Set 3) comparing the relative attractiveness of autoclaved and non-autoclaved infusion [47,76]. The experiment followed the same experimental procedures as described above. After filtering the infusion through a cloth on the day of experiment, the infusion was split in two equal volumes and half autoclaved at 120°C for 20 min to kill bacteria potentially involved in releasing oviposition semiochemicals [47,77] and to reduce the amount of volatile chemicals from the solution whilst maintaining the colour and turbidity of the infusion. After autoclaving, the infusion was left to cool to ambient temperature before setting up the cage bioassays. The oviposition choice of individual gravid females was evaluated for six-day old soil infusion *versus* lake water, autoclaved six-day old soil infusion *versus* lake water and for autoclaved *versus* non-autoclaved six-day old infusion. An equal number of cages were set up where both cups in a cage contained lake water and were randomly labelled as control and test cup.

In order to confirm that autoclaving sterilized the infusion, samples (1 ml) of both infusions were taken during each experimental round for bacterial cultures. Samples were serially diluted (ten-fold) two times in distilled water. After dilution, 100 μl of each of the ×1 (undiluted), ×10^−1^ and ×10^−2^ dilutions was spread separately onto the surface of duplicate Lysogeny Broth (LB) agar-plates (LB Lennox-Fisher Scientific) [78]. Plates were incubated overnight at 30°C and the presence of colonies recorded.

The same physical and chemical parameters were measured for the autoclaved infusion as described above for the non-autoclaved pellet and soil infusions.

### Is the choice of the gravid female influenced by her olfactory memory of her larval habitat?

A fourth set of experiments (Table 1, Set 4) was designed to assess the possibility that a gravid female’s choice for an oviposition site might be influenced by her olfactory memory of her larval habitat, as has been suggested for culicine species [4,79].

To test this, approximately 2,000 *An. gambiae s.s.* eggs were dispensed in 1.5 l of two-day old pellet infusion and another 2,000 eggs in lake water and reared under the same conditions to the adult stage. The infusion and lake water in the rearing pans was replaced every two days with fresh two-day old infusion or lake water until all surviving larvae pupated. Larvae were fed with Tetramin® fish food twice daily following routine insectary procedures. Pupae were collected in a cup with 100 ml of rearing water and placed in 30×30×30 cm cages for emergence. Gravid females for cage bioassays were obtained as described above.

Dual-choice cage bioassays were carried out in parallel with gravid *An. gambiae s.s.* reared in the infusion and gravid *An. gambiae s.s.* reared in lake water. A single mosquito was offered a choice between six-day old pellet infusion or lake water. Forty-five replicates were set up in parallel for both treatment groups as described above.

### Sample size considerations

The sample size (number of responders) in the four sets of cage experiments differed for a number of reasons. Due to adverse climate conditions affecting the mosquito supply during the pellet infusion bioassays, the production of colony-reared mosquitoes was low. Nevertheless, two-sample comparison of proportions power calculation showed that 66 responders in each arm in the pellet infusion bioassays (Table 1, Set 1) was sufficient to detect a 23% increase or decrease in the proportion of eggs laid in the treatment compared to the lake water only experiment with 80% power at the 5% level of significance. The effect of the pellet infusion observed on oviposition response was much stronger than 23%. In the soil infusion experiments (Table 1, Set 2 and Set 3), a minimum of 150 responders in each arm was analysed. This was sufficient to detect a 15% increase or decrease in the proportion of eggs laid in the treatment as compared to the lake water only experiment at the same power and significance level. This level of accuracy was deemed appropriate for investigating significant behavioural cues affecting the oviposition choice. The evaluation of olfactory memory required the mosquitoes to be reared in pellet infusion where larval mortality was nearly 98%. Therefore, only 45 females could be tested, out of which only 31 and 37 responded in the two arms (Table 1, Set 4). The hypothesis for this experiment was that the preference of gravid females could be changed and therefore at least double the proportion of eggs laid in pellet infusion by infusion-reared females as compared to the lake water-reared females. With 31 responders in each arm the experiment was powered (80%) to detect a change in the proportion of 35%.

### Statistical analyses

All data were analysed in R statistical software version 2.13.1 [80]. The one sample proportion test function was used to estimate the 95% confidence intervals (CI) for the proportion of larvae surviving in pellet infusion, soil infusion and lake water. Pupation time of larvae exposed to different treatments was calculated using the following formula: (A×1) + (B×2) + (C×3)…(G×10)/Total number of pupae collected, where A, B, C…G are the number of pupae collected on day 1, 2, 3 to 10. Dual-choice, egg-count bioassays were analysed using generalized linear models (glm-function) with a quasibinomial distribution fitted to account for the overdispersion. In the first three sets of experiments the proportion of eggs laid in test cups in the cages with equal treatments (lake water in both cups) were compared with the proportion of eggs laid in test cups in cages with two different treatments. It was hypothesized that gravid females presented with an identical treatment lay in both cups in an approximately equal proportion (p = 0.5). The statistical analysis aimed to reveal if the test treatment of interest (e g, infusions of different age) received an increased or decreased proportion of the total number of eggs laid as compared to the lake water only treatment. Therefore, the treatment choice (e g, lake water only cages, cages with infusion *versus* lake water) and the round of experiment were included as fixed factors to analyse their impact on the outcome (proportion of eggs laid in test cup). A similar analysis was used for the fourth set of experiments to compare the proportion of eggs laid in test cups (pellet infusion) by gravid females that were reared in hay infusion during their larval development, compared to the proportion of eggs laid in test cups by gravid females that were reared in lake water. The mean proportion of eggs laid in test cups in different treatments and their 95% CIs were calculated as the exponential of the parameter estimates for models with no intercept included. Similarly, multiple comparisons of treatments were calculated based on the model parameter estimates.

## Results

### Gravid *Anopheles gambiae sensu lato* females discriminate between habitats when searching for an oviposition site

**Figure 2:**
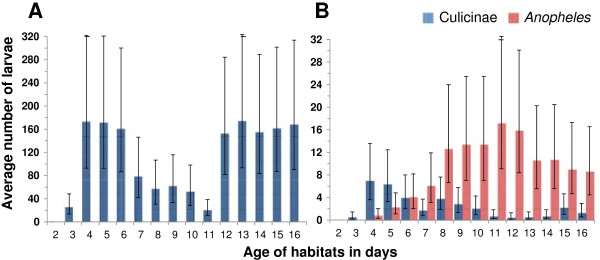
**Natural colonization of artificial habitats.** Daily average of early instar larvae in **(A)** pellet infusions; **(B)** soil infusions.

Mosquitoes oviposited in the artificial ponds shortly after they had been set up since early instar larvae were found from day 3 and larvae hatch approximately 24–48 hours after eggs are laid. Ponds with pellet infusion were colonized exclusively and in high densities by culicine mosquitoes. Not a single *Anopheles* larva was detected over the 16-day observation period. In sharp contrast, early *Anopheles* instar were consistently found from day 3 to day 16 in the soil infusion ponds (Figure 2). Based on the pattern of larval abundance, peak oviposition occurred six to ten days after setting up the ponds*. Anopheles* nearly always occurred in higher densities than culicines. *Anopheles* larval densities are naturally relatively low in the study area, with approximately one to three larvae/dip in natural habitats [17]. In the present study an average of ten (95% CI 5–18) early instar larvae/dip was recorded, indicating that the soil infusion ponds were a highly favourable habitat.

All pupae collected from the artificial habitats belonged to the *An. gambiae* complex. PCR-based species analysis revealed that nearly all the wild *An. gambiae s.l.* were *An. arabiensis* (98%, 49/50).

### The female’s oviposition choice benefits the survival of her offspring

**Figure 3:**
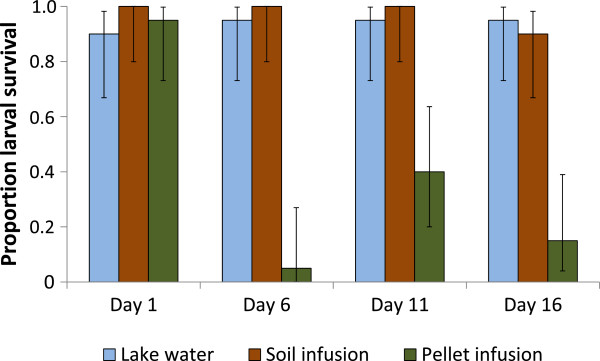
**Survival of *Anopheles gambiae sensu stricto *larvae to the pupal stage kept in different infusions or lake water.** Error bars show 95% confidence intervals.

*Anopheles gambiae s.s.* larvae survived equally well in soil infusion and lake water irrespective of the age of the infusion. In contrast, larvae placed in pellet infusion only survived in the one-day old infusion in similar numbers but survival was reduced by over 60% (p < 0.001) in pellet infusions six days and older compared to lake water or soil infusions of the same age (Figure 3). Mean pupation time for survivors did not significantly differ between treatments or ages of the infusion and was on average 7.5 days (95% CI 6.6-8.3).

### Gravid female *Anopheles gambiae sensu stricto* show avoidances and preferences when selecting an oviposition site

**Figure 4:**
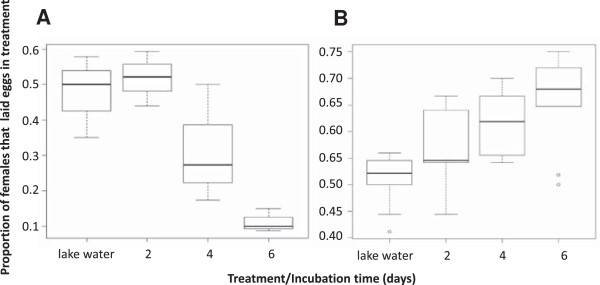
**Proportion of gravid *Anopheles gambiae sensu stricto *laying eggs in infusions of different ages compared with control water. (A)** Pellet infusion experiment; **(B)** soil infusion experiment.

Figure 4 shows the median response rate of the gravid females to the test cup in pellet infusion and soil infusion experiments. An approximately equal proportion of females laid eggs in test and control cups when an equal choice of lake water was provided. Fewer females laid their eggs in pellet infusion as it aged, whilst for soil infusion the opposite was the case with an increasing proportion of females laying eggs in soil infusion as it aged.

**Figure 5:**
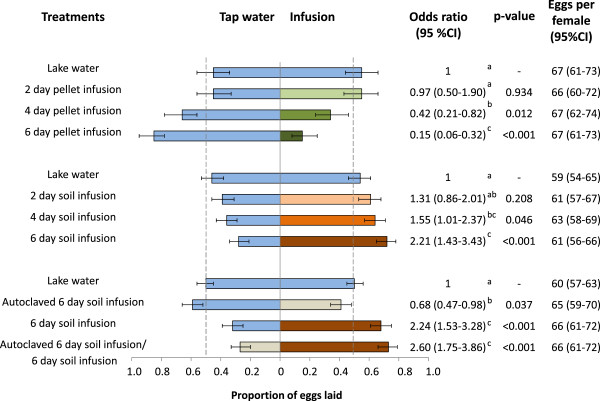
**Oviposition response of caged *Anopheles gambiae sensu stricto *to pellet (Set 1)and soil (Set 2) infusions of different incubation times and non-autoclaved and autoclaved 6 day soil infusion (Set 3).** Multiple comparison of treatments: treatments denoted with the same letter are not significantly different.

Similar results were seen for the proportions of eggs laid although the distribution of eggs between the two equal choices of lake water was slightly skewed (Figure 5, Set 1, lake water control cup 0.45 (95% CI 0.34-0.56) *versus* lake water test cup 0.55 (95% CI 0.44-0.66)) though not significantly different from 0.5. The distribution of eggs between lake water and two-day old pellet infusion did not significantly differ from the distribution between the two cups with lake water only. However, pellet water became unattractive from day 4 (Figure 5, Set 1). It was 6.7 times less likely for an egg to be laid in the test cup in the treatments that contained six-day old pellet infusion *versus* lake water than it was when both cups contained lake water. During the experiment with soil infusions a similarly skewed distribution in the proportion of eggs laid in the two cups with lake water was observed due to chance alone. (Figure 5, Set 2). In contrast to the pellet infusion, larger proportions of eggs were laid in the test cups with increasing age of the soil infusion. An egg was more than twice as likely to be laid in the test cup in the treatments that contained six-day old soil infusions compared with lake water than it was when both cups contained lake water (Figure 5, Set 2).

On average individual females laid 63 eggs (95% CI 60–65) (Figure 5) irrespective of the experiment and treatment. Notably, 18% (95% CI 11-26%) of gravid females laid eggs in both cups provided in a cage, a behaviour known as skip oviposition in other mosquito species [81] but rarely reported for *An. gambiae s.s*. [82]. The average number of eggs laid by skip-ovipositing females and by females that chose only a single cup was similar. Whilst the percentage of skip-ovipositing females was similar in all treatments with two equal lake water choices and in all soil infusion treatments, this behaviour was affected by the pellet infusion. Only a few *An. gambiae s.s.* females skip-oviposited in the four- and six-day old pellet infusion treatments (6%, 95% CI 3-9%).

Pellet and soil infusions differed in key physical and chemical parameters. All pellet infusions had a strong smell to the human nose, were more or less transparent and had a slightly green colour but differed little in appearance compared with lake water in the oviposition cups. Correspondingly, turbidity levels were low. In contrast, soil infusions did not have any smell to the human nose, were light brown in colour and turbid, providing a strong visual contrast to the lake water. Pellet infusions were also characterized by relatively high conductivity, low pH and oxygen deprivation. In contrast the soil infusions’ conductivity was approximately half that of pellet infusions, was saturated with dissolved oxygen and had a higher pH (Table 2). The variability of these measures between infusions of different incubation times within a treatment group was relatively low and does not appear to explain the differences in the behavioural responses. The only factor that changed over time was turbidity in the soil infusion and notably the most preferred six-day old infusion was less turbid than the others.

**Table 2:** Physical and chemical properties of pellet and soil infusions

**Parameter**	**Oviposition substrates in choice experiments**							
	**Lake water**	**Pellet infusions**			**Soil infusions**			
		**2 days**	**4 days**	**6 days**	**2 days**	**4 days**	**6 days**	**Auto-claved**
Turbidity (NTU)	1	22	17	25	222	97	73	137
	(0.6–1.4)	(20–23)	(14–19)	(20–29)	(197–248)	(92–102)	(61–84)	(108–166)
Conductivity (uS/cm)	107	477	553	543	173	207	237	266
	(105–110)	(462–491)	(547–559)	(532–555)	(171–176)	(203–212)	(233–242)	(258–274)
Dissolved oxygen (mg/l)	4	0.3	0.7	0.3	5.3	6	6.3	4.8
	(2.7–5.3)	(0.21–1.4)	(0.9–1.4)	(0.2–0.4)	(5.0–5.6)	(5.7–6.3)	(6.0–6.6)	(4.6–5.1)
pH	8.1	6.3	6.7	7.4	7.7	7.9	8	8.9
	(7.9–8.1)	(6.2–6.3)	(6.5–6.9)	(7.3–7.5)	(7.6–7.7)	(7.8–7.9)	(7.9–8.0)	(8.8–8.9)
Ammonium (mg/l)	0			5			0	0
Nitrate (mg/l)	10			10			10	10
Nitrite (mg/l)	0			0			0.025	0.025
Phosphate (mg/l)	0			3			1.5	1.5
Carbonate hardness (mmol/l)	0.1			3.9			2.7	2.5
Total hardness (mmol/l)	0.1			1.5			0.7	0.7

The increased carbon and total hardness of the pellet infusion corresponded with the increased conductivity levels and the high ammonium and phosphate levels compared with the soil infusion (Table 2).

### Chemical cues from the infusions are responsible for the oviposition choice in cage bioassays

Since soil infusions differed in appearance from the lake water, an additional set of experiments was carried out to evaluate whether the attractiveness of this infusion was due to visual cues. Cage experiments with two equal choices of lake water confirmed an equal distribution of eggs between control and test cup. Notably, when gravid females had a choice between lake water and autoclaved soil infusion, the lake water was preferred. The oviposition preference for six-day old soil infusion compared with lake water was also confirmed in this set of experiments with nearly identical odds ratios as before of 2.2. The preference for the six-day old infusion was corroborated when given a choice between autoclaved and non-autoclaved infusions of similar colour and turbidity (Figure 5, Set 3).

Results from this set of experiments suggest that chemical cues are involved in the oviposition responses observed. If the preference for the six-day old soil infusion over lake water was based on turbidity and/or colour of the infusion alone, a similar response in the choice tests with autoclaved *versus* non-autoclaved infusion should have been seen as in the choice tests with lake water *versus* autoclaved infusion. Due to the slight avoidance of the autoclaved infusion the odds of finding an egg in the non-autoclaved six- day old infusion should have been approximately 1.3 (decreased choice for autoclaved infusion by 30% or increased choice of fresh infusion by 30%). Nevertheless, the remaining odds of 2.2 can only be explained by chemical cues being either involved in attracting the female from a short distance or stimulating the female to lay eggs on contact with water. Physical and chemical water parameters were similar for autoclaved and non-autoclaved soil infusions. Bacteria cultures from autoclaved infusions confirmed that samples did not contain any bacteria that could grow on LB plates as opposed to the non-autoclaved infusion where colonies of at least three different morphologies were observed.

### The oviposition choice of the gravid female is not influenced by her olfactory memory of her larval habitat

**Figure 6:**
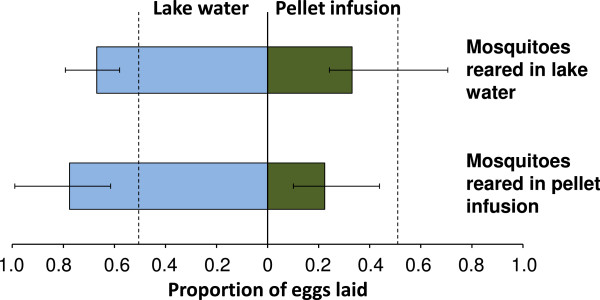
**Egg laying responses of *Anopheles gambiae sensu stricto *** **reared in lake water or in pellet infusion to lake water and pellet infusion (Set 4).**

Rearing *An. gambiae s.s.* in two- to four-day old pellet infusion did not alter their oviposition response towards the infusion (p = 0.392). Gravid females reared in lake water and gravid females reared in pellet infusion show an equally strong avoidance of the six-day old pellet infusion provided in choice experiments (Figure 6, Set 4).

## Discussion

The results confirm that wild and caged *An. gambiae s.l.* females discriminate (defined as recognition and understanding of the difference between two things [83]) between potential aquatic habitats for oviposition and make clear choices when presented with contrasting oviposition media. These choices benefit the survival of the offspring. Although the experimental design does not allow a conclusion whether the stimuli acted over a distance (attractants and repellents [84]) or on contact with the oviposition medium (stimulants and deterrents [85]), it could be demonstrated that the choice of breeding site is guided by both avoidance and preference. However, the exclusive way in which the artificial ponds were chosen in the field, where they were set up relatively close to each other, suggest that these characteristics were detected by both culicines and anophelines from a distance rather than on contact.

Muddy water has previously been suggested to increase oviposition response of gravid *An. gambiae s.s.* in cages when offered together with clear water [41], however in this study cage experiments could not confirm this observation. The difference in turbidity between lake water and infusions cannot explain the avoidance and preference observed at short range in the cages. Two-day and six-day old pellet infusions did not differ in their turbidity but significantly differed in the oviposition response they received. Similarly, all soil infusions should have elicited equally strong responses from gravid females if turbidity was an important oviposition cue at short range. On the contrary, the six-day old soil infusion remained equally preferred for oviposition when tested against a turbid and autoclaved infusion than when tested against clear lake water. The results suggest that chemical and not visual cues were responsible for the responses observed in the cage experiments. The previously published preference of muddy water [41] may have been based on chemical cues associated with the muddy water which was taken from a natural habitat. However the possibility that visual cues played a role in the selection of oviposition sites by wild mosquitoes in field experiments especially when searching for water bodies from a distance cannot be entirely excluded.

It is likely that the chemical cues used by mosquitoes to avoid pellet infusions and to prefer soil infusions were at least partly of microbial origin, which is supported by the lack of attraction to the autoclaved soil infusion compared to clear lake water. It is most likely that the observed oviposition choices as well as the larval survival were rooted in the water quality of the habitat and consequently the associated micro-organisms and chemicals in the water. The physical and chemical water parameters measured for the two infusions suggest that they represented aquatic habitats in different stages of decay. Contrary to the expectation at the onset of the experiment, pellet infusions created with increasing age a habitat type in a severe state of decomposition. High ammonia and phosphate levels are characteristic of recently inundated organic material [86]. The odour of the pellet infusion is associated with fermentation of organic material by facultative and anaerobic bacteria. This leads to depletion of the oxygen supply and a decrease in pH as a result of accumulation of organic acids in the water [87-89]. The soil infusion had the characteristics of a less nutrient-rich habitat containing relatively little organic matter. This limits the removal of oxygen by aerobic heterotrophic micro-organisms and hence the water column will stay aerobic [89].

Anaerobic fermentation products of organic matter have been previously shown to be highly attractive to a number of gravid culicine species such as *Culex stigmatosoma* (formerly *peus*) [87], *Culex quinquefasciatus*[33,65,90-92], *Culex pipiens*[64], *Aedes aegypti*[77,93] and *Aedes albopictus*[94,95]. These infusions have been associated with a range of bacteria such as *Aerobacter aerogenes, Pseudomona aeruginosa*, *Bacillus cereus*[96-98] and volatile chemicals produced by them including 4- methylphenol, 3-methylindole, carboxylic acids and methyl esters [33,39,99,100]. It is likely that similar factors were responsible for the strong repellent/deterrent effect on gravid *An. gambiae s.l.* females.

Most stagnant water bodies will show increasing signs of decomposition over time but the speed and extent of this will depend largely on habitat quality [101]. Therefore, it is argued that habitat age or permanence alone is not a good predictor for the oviposition response of *An. gambiae s.l.* as has been suggested [45]. For example, the content and input of organic matter, source of water and frequency of fresh water inflow will affect the composition of the biotic community and chemical and physical characteristics of an aquatic habitat [86,89,101]. This might explain why in some environments semi-permanent and permanent habitats are just as well colonized as temporary habitats traditionally thought to be the preferred *An. gambiae s.l.* habitats [13,17,67]. Habitats made of pellet infusion were avoided by *Anopheles* from an early habitat age, whilst interestingly, the highest preference of the soil infusion was recorded on day 6 in the laboratory and between day 6 and 10 in the field after the habitats were well established, contradicting the idea that *An. gambiae s.l.* is a pioneer species colonizing temporary habitats immediately after their occurrence [13].

Typically, it is reported that *An. gambiae s.l.,* although largely a generalist, is not found in heavily polluted waters [19,102]. Hancock [103] further observed that *An. gambiae s.l.* avoided water with a low pH when it was also accompanied with high organic matter content. Addition of freshly cut vegetation (i e, grass cuttings) to aquatic habitats has also been shown to prevent the larval development of *An. gambiae s.l.*[16]. The results from the experiments with pellet infusions support these observations. On the other hand, there have been recent reports of *An. gambiae s.l.* colonizing polluted habitats especially in urban areas [104-106]. Clearly, the degree of avoidance or acceptance of a polluted habitat by *An. gambiae s.l.* depends on the extent and nature of pollution [19]. Results show that two-day old pellet infusions were not rejected by *Anopheles* and even four-day old infusions still received a considerable proportion of the oviposition responses despite their adverse water characteristics. This supports the idea that *An. gambiae s.l.* has a very high tolerance level of what they accept as oviposition sites, especially in the absence of better alternatives in close vicinity as is often the case in urban environments and in contrast to the here presented field experiment where good habitats were offered right next to the unfavoured ones.

Importantly, *An. gambiae s.l.* appears to have an innate propensity to avoid specific chemical cues that were emitted from the pellet infusion. Rearing *An. gambiae s.s.* from egg to pupae in this infusion did not alter this behaviour. Gravid females that had experienced the pellet infusion during larval development avoided the infusion for oviposition as much as the females that had no prior experience of it. This suggests that the environment in which *An. gambiae s.s.* develop as larvae does not determine the preferred oviposition site when they return to lay eggs. This is in contrast to published work on *Cx. quinquefasciatus* where it was demonstrated that rearing the larvae in an infusion made from guinea-pig faeces cancelled their innate preference for a hay infusion [4].

The cage bioassays with individual gravid females allowed a number of interesting observations that are rarely reported since the majority of studies with *An. gambiae s.s.* are done with groups of mosquitoes where the actual number of females laying per cage is unknown [41]. The occurrence of skip-oviposition in gravid *An. gambiae s.s.* and how this is affected by chemical cues was demonstrated. Furthermore, the design revealed that the mean number of eggs laid per female in a cage was similar irrespective of the experiment and treatment; only the distribution between cups differed when two different choices were presented. This indicates that gravid females did not retain their eggs in the presence of an unfavoured substrate when they were offered a suitable alternative choice. It also shows that the preferred soil infusion did not stimulate individual females to lay more eggs than they would do in lake water. Testing individual females also excludes potential aggregation effects. Whilst from the field experiments it might have been possible that gravid females selected habitats that already received eggs from conspecific females, cage bioassays with individual females showed the same avoidance and preference behaviour as observed in the field, confirming that conspecifics alone cannot explain the observed choice.

The potential involvement of microbial activity in breaking down organic matter and producing semiochemicals that impact on the oviposition responses of gravid *An. gambiae s.s.* was deduced partly by the lack of attraction of *An. gambiae s.s.* to a sterile soil infusion. However, this must be interpreted with caution since autoclaving the infusion might not only have killed the microbes but affected the chemistry of the resulting infusion, possibly altering the response of gravid mosquitoes by chemical changes rather than biological changes [77].

Batch-to-batch variations were recorded in the response of gravid mosquitoes to the infusions, resulting, for example, in some rounds showing a high preference and other rounds only a moderate preference for the soil infusion. This variation can be attributed to differences in the quality and amounts of odorants released from the infusions and stochastic events. Fresh infusions were prepared for every test round with different batches of pellets and soil. Especially, for the soil it is highly likely that there were differences in the soil condition as well as differences in the species composition of the microbial community associated with the natural materials over time. It has been shown previously that natural infusions can be an inconsistent source of odorants for oviposition site-seeking mosquitoes and therefore every batch needs to be verified to be behaviourally active before it can be used for subsequent experiments [77]. Ideally, if info-chemicals were to be used for monitoring and/or controlling gravid malaria vectors, specific chemically defined oviposition cues would be preferred over natural infusions to ensure a consistent response in gravid females either pushing them away from human population [107,108] or pulling them towards a gravid trap [38,109].

Whilst the observed avoidance behaviour towards the organically rich pellet infusion was strong and in the same range as reported for other species in response to unfavourable chemical cues [107,108,110], the observed preference in the cages for the soil infusion was relatively weak and it is questionable whether it could compete with other suitable habitats from a larger distance. Nevertheless, consistent response derived from over 150 replicates in two experiments likely represents a genuine effect. Further investigations are in progress to characterize the bacteria communities associated with the infusions and the volatile chemicals emitted from the infusions and detected by gravid *An. gambiae s.s.* using gas-chromatography coupled to mass-spectrometry and coupled gas chromatography-electroantennogram detection.

It must be cautioned that not all soils and all rabbit food pellets will lead to the same physical and chemical parameters as the infusions presented here. Therefore the two infusions of this study only serve as specific examples for two highly contrasting media. Further work is needed to screen other soil samples to see if the observed response is a response common for all soil infusions prepared under standard conditions and if the same bacteria and chemical profiles can be detected, or, which is more likely, that there are significant differences depending on the source of the soil.

## Conclusion

This work illustrates that a gravid *An. gambiae s.l.* female selects a suitable habitat for oviposition using chemical cues from water bodies. It furthermore emphasizes that natural infusions can be used to manipulate the oviposition behaviour of *An. gambiae s.l.*. Soil infusions have the potential to be used to bait gravid traps for the collection of *An. gambiae s.l*., although further work must be implemented to elucidate whether the observed preference was based on the specific soil type tested or whether similar responses can be achieved with any soil. The low *An. gambiae s.l.* catching efficacy reported for gravid traps operationally used for *Culex* and *Aedes* monitoring might partly be explained by the infusions routinely used in these traps, i e, fermented hay infusions, rabbit food pellet and cow manure infusions [64-112]. The identification of the chemicals responsible for the preference of the soil infusion might be exploited to bait gravid traps specifically for the collection of *An. gambiae s.l.*

## Competing interests

The authors declare that they have no competing interests.

## Authors’ contributions

UF, JL, MH-V and SWL conceived the idea for this research and developed the experimental design. MH-V developed all protocols and implemented the experiments. MH-V and UF analysed the data and wrote the first draft of the manuscript. All authors contributed to the final draft, read and approved the manuscript.
